# A Review of Image Sensors Used in Near-Infrared and Shortwave Infrared Fluorescence Imaging

**DOI:** 10.3390/s24113539

**Published:** 2024-05-30

**Authors:** Banghe Zhu, Henry Jonathan

**Affiliations:** The Center for Molecular Imaging, The Brown Foundation Institute of Molecular Medicine, The University of Texas Health Science Center, Houston, TX 77030, USA

**Keywords:** image sensors, near-infrared, shortwave infrared, fluorescence imaging, CCD, CMOS, measurement sensitivity

## Abstract

To translate near-infrared (NIR) and shortwave infrared (SWIR) fluorescence imaging into the clinic, the paired imaging device needs to detect trace doses of fluorescent imaging agents. Except for the filtration scheme and excitation light source, the image sensor used will finally determine the detection limitations of NIR and SWIR fluorescence imaging systems. In this review, we investigate the current state-of-the-art image sensors used in NIR and SWIR fluorescence imaging systems and discuss the advantages and limitations of their characteristics, such as readout architecture and noise factors. Finally, the imaging performance of these image sensors is evaluated and compared.

## 1. Introduction

Over the past 25 years, a new technique called near-infrared (NIR) fluorescence imaging has been developed and translated into the clinic. NIR fluorescence imaging utilizes the wavelength ranges from 690 nm to 900 nm, called the NIR window, within which the hemoglobin and water in tissues exhibit low absorption such that NIR light can penetrate deeply into the tissue, allowing the imaging of large-volume tissues, such as the breast and brain [1]. Although a variety of NIR fluorophores are commercially available, which include dyes such as Cy 5.5, Cy 7, quantum dots (QDs), IRDye 800CW, Indocyanine Green (ICG), and others [2,3], ICG is the only NIR-excited fluorophore used clinically for applications like NIR fluorescence angiography in coronary [4], neurosurgical, and vascular surgeries [5], and applications like NIR fluorescence lymphangiography in mapping the lymphatic vasculatures and identifying the sentinel lymph nodes in surgical oncology [6], and many others such as cancer localization and surgical margin assessments [7].

Recently, NIR fluorescence imaging has been expanded into the shortwave-wavelength infrared light (SWIR, 1.0–2.0 μm), called SWIR fluorescence imaging [8]. In the longer wavelengths, the tissue light scattering is significantly reduced, the autofluorescence becomes negligible, and the total absorption from tissue components is comparable to that in the NIR window. These factors result in high-resolution imaging at greater penetration depth than is achievable in the NIR window. Since in vivo SWIR fluorescence imaging is a relatively new field of research, only a few SWIR fluorophores were developed, including single-walled carbon nanotubes (SWNTs) [9,10], QDs [11], rare earth-doped nanoparticles [12], polymeric nanoparticles [13], ruthenium (II) metallacycle [14,15], and small-molecule water-soluble dyes [16]. However, none of these SWIR fluorescent agents have been approved for clinical applications. Fortunately, ICG exhibits fluorescence emission in the SWIR window and has been used to perform state-of-the-art SWIR in vivo imaging [17], including intravital microscopy, noninvasive real-time imaging in blood and lymph vessels, imaging of hepatobiliary clearance, and molecularly targeted in vivo imaging [18].

In order to translate the new fluorescence imaging agents into the clinic, a micro dose of fluorescent agent needs to be tested in clinical trials to evaluate its toxicity. This requires the fluorescence imaging devices to be sensitive enough to detect emitted fluorescence signals from trace dose agents in tissues. With well-selected excitation light sources such as laser diodes and light-emitting diodes and filtration schemes optimized through the introduction of collimation optics and the high density of optical filters, the imaging performance of a device is ultimately determined by the image sensors [19,20,21,22]. The image sensors for the majority of cameras are commonly made of light-sensitive silicon (Si) material in addition to gallium–arsenide (GaAs) material in intensified image sensors. However, Si and GaAs sensors are not sensitive at wavelengths of >1000 nm, excluding the possibility of SWIR imaging [23]. Instead, SWIR cameras use semiconductor alloys with narrower bandgaps such as indium–gallium–arsenide (InGaAs) and QDs, which have a high quantum efficiency (QE) in the SWIR window [24].

To date, NIR and SWIR fluorescence imaging have been discussed in various review papers and studies. For example, Li et al. [25] examined the different fluorophores and imaging systems used in the SWIR window as well as their importance and the benefits they can bring to biomedical applications. Another article, by Carr et al. [18], discussed how the limited availability of detection technology and FDA fluorophores with peak emissions in the SWIR region have limited the use of SWIR imaging in clinical applications. In comparison, this review article focuses more on the sensors involved in the NIR and SWIR imaging process, rather than the fluorophores or contrasting agents necessary for image processing.

## 2. The Types of Image Sensors

### 2.1. Image Sensors Used in NIR Fluorescence Imaging Systems

The widely used image sensors in NIR fluorescence imaging systems include the charge-coupled device (CCD), electron-multiplying CCD (EMCCD), complementary metal–oxide–semiconductor (CMOS), and the intensified CCD (ICCD) and CMOS (ICMOS).

#### 2.1.1. CCD Cameras

CCDs are semiconductor image sensors that can convert light into electrical signals. CCDs are composed of an array of photodiodes (light-sensitive pixels) that are electrically sensitive, so that when light is shone on them, they generate and store electrons. The number of electrons contained within each pixel correlates to the number of photons illuminating the pixel. The electron (charge) is then “read out” and converted digitally into a numerical value [26]. This set of steps is repeated for each pixel within a CCD to create a picture of the scene.

**Figure 1:**
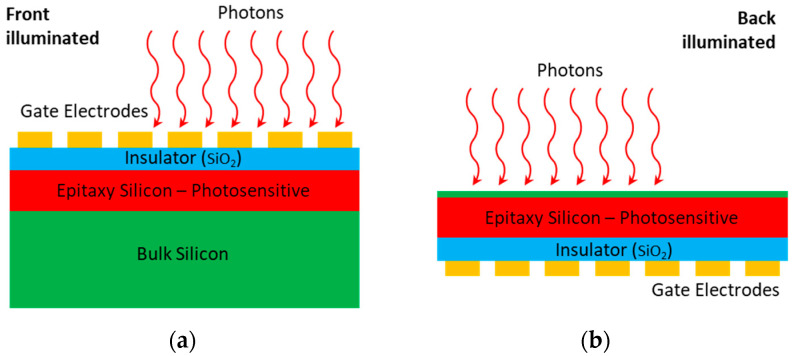
Schematics of (**a**) front-illuminated CCD (FCCD) and (**b**) back-illuminated CCD (BCCD). The FCCD is designed with the polysilicon gate located at the front, the photodiodes at the back, and the wiring in between. The BCCD is similar except its polysilicon gate is located at the back and some of the bulk silicon substrate is removed.

CCDs can be classified into two separate forms, the front-illuminated CCD (FCCD) and back-illuminated CCD (BCCD), based on their architectures [19]. FCCDs are designed with a similar structure to the human eye, with the polysilicon gates at the front, the photodiodes at the back, and the wiring in between. A simple schematic of an FCCD is shown in Figure 1a. In this configuration, incident photons are partially absorbed or reflected by the gate electrodes before reaching the photodiodes, resulting in a relatively low QE. BCCDs have the same elements, but by rearranging the gate structure to the back of the photosensitive area of the CCDs in addition to removing some of the bulk silicon substrate through mechanical or chemical etching techniques (Figure 1b), BCCDs have more than a two-fold improvement in QE over their front-illuminated counterparts. However, a drawback of the BCCD is that the longer wavelengths of light can pass through the photosensitive region and reflect off the surfaces to create interference fringes, which contaminate the normal spectrum.

There are four common readout architectures that are used in CCD image sensors, which include the full frame (FF), frame transfer (FT), interline transfer (IT), and frame-interline transfer (FIT) [27]. An FF CCD is primarily used for applications that require long exposure times without electronic shuttering while having a high QE value and a 100% fill factor. An FT CCD is a similar readout architecture compared to the FF CCD except that it has a shuttering function. The third type of readout architecture for CCD image sensors is the IT CCD. An IT CCD uses full electric shuttering during video recordings and still captures moving objects. One of the key performance factors that affect IT CCDs is smear, which is generated when a charge is moved into the CCD quickly. Lastly, FIT CCDs are a modified form of IT CCDs that include shuttering in order to eliminate smear from the image. In general, CCDs employ a single A/D converter for readout for the entire array, which leads to the optimal noise characteristics.

One advantage of CCDs is that they require minimal pixel overhead, allowing the creation of image sensors with relatively small pixel sizes. Another advantage of CCDs is how they are able to retain their performance quality even when their pixel size is scaled down [28]. Other general advantages of CCD cameras include their superior dynamic range, low fixed-pattern noise (FPN), and high sensitivity to light [29]. Although CCDs have some useful advantages, they still have some disadvantages that require consideration before use. The main disadvantages to consider are that CCD technology is very energy draining and that when using CCD image sensors, other functions cannot be integrated onto the same chip as the image sensor [29]. CCD technology has been widely adopted in a large number of NIR fluorescence imaging systems. Some of the most prominent NIR fluorescence imaging systems include fluorescence molecular tomography [30] as well as NIR fluorescence-guided surgery imaging systems such as the Storz D-Light and SPY imaging systems [31].

#### 2.1.2. EMCCD Cameras

**Figure 2:**
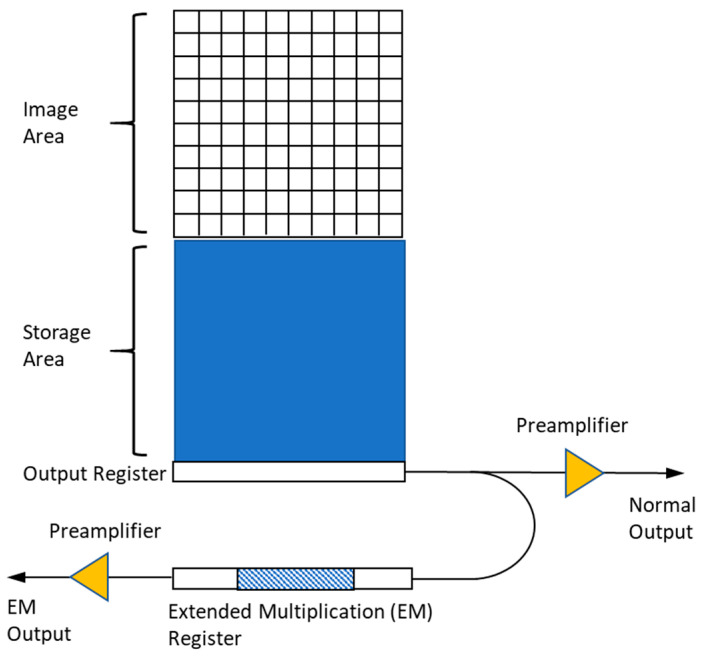
Schematic of EMCCD. It consists of a CCD with an additional electron-multiplying (EM) register located between the end of the normal serial register and the output amplifier.

EMCCDs are a variant of CCDs that introduce an additional electron-multiplying (EM) register between the end of the normal serial register and the output amplifier to elevate the electron signal greatly above the read noise floor for ultra-low-light detection [32,33]. The EM register consists of several hundred stages that use higher-than-typical CCD clock voltages (up to 50 V) to accelerate the photoelectrons and generate the secondary photoelectrons via the impact ionization process, as shown in Figure 2. The degree of multiplication gain can be controlled by varying the clock voltages applied to the EM register, with an EM gain of >1000×. Due to this photoelectron amplification mechanism, EMCCD cameras are capable of detecting a single photon with sub-electron readout noise at high frame rates.

There are several noise sources that can significantly reduce the performance of EMCCD cameras, such as shot noise, dark current, and readout noise [34]. Shot noise is a fundamental property of the quantum nature of light and appears due to the change in the number of photons emitted by an object. There is always some form of shot noise in imaging systems as there is currently not a method to reduce it completely. Dark current is a noise source that is made by thermally generated electrons in the Si substrate of the CCD portion of the image sensor. Lastly, readout noise is made by the electronic circuitry, which converts the charge within each pixel into a digitized light-intensity proportional number that is displayed in the image. When the accumulated charges are transferred onto the CCD chip, some electrons can be left behind or jump ahead, causing noise to occur in the image. The prominent disadvantage of using EMCCD cameras is that any noise generated by the both the dark current and the charge-transfer process across the sensor area before reaching the gain register would be multiplied [35]. This is the main reason why EMCCDs have to be cooled at relatively low temperatures, at around −70 °C, compared to other image sensors. Thus, it is important to consider the noise performance of an EMCCD device before practical use, even though it theoretically has high capabilities. The EMCCD cameras are widely employed for conducting low-light imaging and applications such as non-invasive NIR fluorescence imaging of the lymphatic vasculatures in small animal models [36,37].

#### 2.1.3. CMOS Cameras

**Figure 3:**
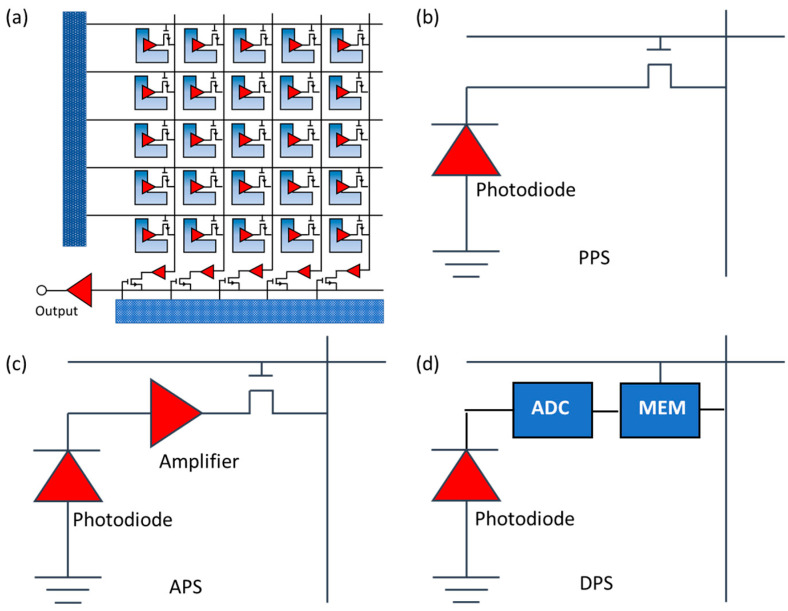
(**a**) CMOS schematic, (**b**) PPS schematic, (**c**) APS schematic, and (**d**) DPS schematic. The CMOS sensors have rows of photodiodes coupled with amplifiers. The design also contains A/D converters for each pixel, which allows the CMOS array to be read out independently and simultaneously. The PPS design contains a photodiode and a single row-select transistor. The APS design consists of a photodiode and either 3 or 4 transistors. The DPS design contains a photodetector, an A/D converter, and a digital memory (MEM) used for temporary storage of data before readout.

Like CCDs, the CMOS sensors are semiconductor image sensors that can convert photons into electrons. CMOS sensors contain rows of photodiodes coupled with individual amplifiers to amplify the electrical signal from the photodiodes [29,38], as shown in Figure 3a. CMOS image sensors can be classified into two different forms based on their pixel architecture, which are the analog pixel sensor and the digital pixel sensor (DPS). There are two sub-types for analog pixel sensors, the passive pixel sensor (PPS) and the active pixel sensor (APS) [28]. The PPS design, as shown in Figure 3b, includes a photodiode and a single row-select transistor. The APS general design, as shown in Figure 3c, consists of 3–4 transistors and a photodiode. APS image sensors are often more popular compared to PPS image sensors. A DPS architecture is a more recent design for CMOS technology. A DPS image sensor consist of a photodetector, an ADC, and a digital memory used for temporary storage of data before the digital readout, as shown in Figure 3d. Some of the advantages that come with using a DPS image sensor include the high-speed readout, and the improved scaling with CMOS technology due to the reduced analog circuit performance demands. The main disadvantage that makes the DPS less popular than the APS is that it requires the use of more transistors per pixel compared to other image sensors, which leads to larger pixel sizes and a lower fill factor.

There are three different types of readout architectures used in CMOS sensors, which include the rolling shutter, snap, and progressive scan readouts [27]. The rolling shutter readout is commonly used for PPS CMOSs. This readout occurs by keeping the reset switch off for a specified number of line periods until a row is chosen for readout. The snap readout is used in APS CMOSs, and is popular because its exposure time is relatively short compared to the readout time. The snap readout begins when a global reset gate is used to reset all pixels at the same time. The charge is then integrated within the photo region for a specific exposure time. The charge is then transferred to the sense node of each pixel at the same time, and readout occurs line by line. The last readout architecture used in CMOS sensors is the progressive scan readout. This method uses a mechanical shutter; after exposure, the charge is read line by line in a similar way to the snap readout.

While CMOS image sensors can be utilized for more versatile applications compared to CCD image sensors, they have more noise sources that reduce their quality. For instance, CMOS image sensors are heavily affected by reset noise, a noise that is generated when pixels are reset, because unlike CCDs, which have correlated double sampling that removes reset noise, CMOS image sensors still retain the noise after it occurs [27]. Another noise that needs to be taken into consideration when using a CMOS image sensor is seam noise. Seam noise causes a difficult-to-remove checkerboard pattern to appear in the image because of the difference in the gain and offset between channels in the multiple readout amplifiers [27]. The use of multiple readout amplifiers, however, does allow CMOS image sensors to be more suitable for high-speed applications compared to other sensors. Recent advances in CMOS fabrication have resulted in very low noise, similar to the amount of noise seen when using CCD image sensors, and as a result, they have been recently adapted into our NIR fluorescence imaging systems and others [39,40,41,42,43,44,45].

#### 2.1.4. Intensified Image Sensors

**Figure 4:**
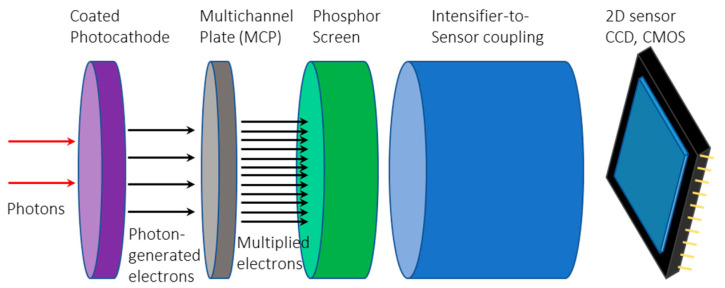
An intensified CCD and CMOS is composed of each respective design plus an additional image intensifier. The image intensifier consists of three main components, a photocathode, a multichannel plate (MCP), and a phosphor screen, where red arrows represent photons and back arrows represents electrons.

Compared to EMCCDs, GaAs-based intensified CCDs (ICCDs) and CMOSs (ICMOSs) have a different multiplication mechanism, in which an image intensifier amplifies the collected image before it is registered by the CCD or CMOS. This mechanism of the image intensifier functions by multiplying the number of incoming photons, which amplifies the incoming light signal, allowing the ICCD and ICMOS cameras to take images in extremely low-light conditions and with extremely short exposure times. The image intensifier was made of three main components, a photocathode, a multichannel plate (MCP), and a phosphor screen, as shown in Figure 4. The low level of incoming light is first converted into electrons at the photocathode, which are then accelerated and amplified at the MCP and, finally, the amplified signal is converted back into a visible green light signal at the phosphor screen ready for the CCD or CMOS acquisition [46]. The generation III intensifiers have greater sensitivity, higher-gain MCPs, and better image quality compared to previous generations [47]. In addition, a thin film of sintered aluminum oxide is attached to the MCP to protect the photocathode from spurious backscattered ions.

There are various considerations that need to take place before using an intensified CCD or CMOS. One factor to consider is the sensitivity of the photocathode, as there are multiple types of photocathodes that cover the spectral range from UV to RGB colors. Another consideration is the type of MCP to use, as they can be separated based on their electron multiplication factor. For instance, a single-stage MCP has an electron multiplication factor of 10^3^, a double-stage MCP has a factor of 10^6^, and a triple-stage MCP has a factor of approximately 10^9^ due to saturation effects. As the stage increases, the optical resolution and the saturation effect decreases, while the SNR for the double-stage MCP is the best, and for the triple-stage MCP, it is the worst. Based on this, the single-stage MCP typically gives the best image quality standard, the second-stage MCP is highly sensitive and optional, and the third-stage MCP is not recommended for use. Lastly, there are three factors to consider before choosing a phosphor screen, which are efficiency, phosphor decay time, and spatial resolution [48]. The two most common phosphor screens when using an image intensifier are the P43 and P46 phosphor screens. The P43 has a high efficiency, long decay time, and a high spatial resolution, while the P46 has a low efficiency, short decay time, and low spatial resolution. Hence, the P46 is better used for fast applications, while the P43 is used when quality is more important. The ICCD camera has been used in frequency-domain NIR fluorescence tomographic imaging systems, where the intensifier is modulated at a radio frequency so that the amplitude and phase information of fluorescence signals can be extracted to improve the accuracy of 3D image reconstruction [49,50]. In addition, both ICCD and ICMOS cameras are widely adopted in NIR fluorescence imaging systems for rapid imaging of lymphatic function in humans [39,51].

### 2.2. Image Sensors Used in SWIR Imaging Systems

The widely used image sensors in SWIR fluorescence imaging systems include the InGaAs-based camera, mercury cadmium telluride (MCT) sensor, Quantum Well Infrared Photodetectors (QWIPs), and QD detectors.

#### 2.2.1. InGaAs

**Figure 5:**
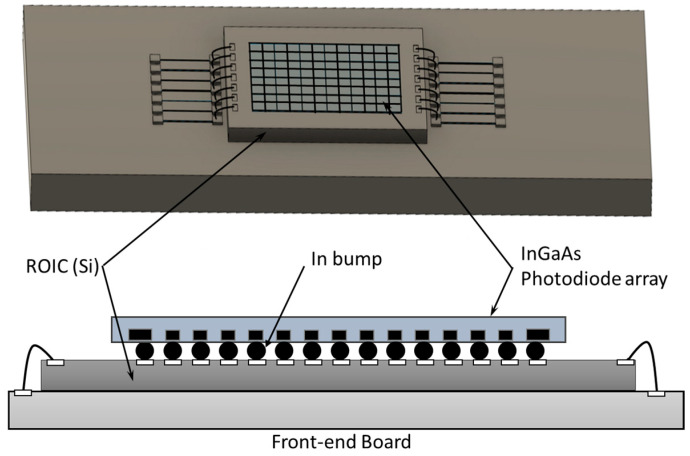
Schematic of InGaAs image sensor. An InGaAs sensor consists of a 2D array, which contains thousands to millions of photodiodes. These photodiodes consist of an indium phosphide (InP) substrate, an InGaAs absorption layer, and an ultrathin InP cap that has an indium bump bonded to a readout integrated circuit (ROIC).

Due to the smaller bandgap of InGaAs, the InGaAs-based cameras have excellent sensitivity in the SWIR region [52,53]. An InGaAs focal plane array is a two-dimensional array containing thousands or even several millions of photodiodes, which comprise an indium phosphide (InP) substrate, an InGaAs absorption layer, and an ultrathin InP cap that has been indium bump-bonded to a readout integrated circuit (ROIC). A diagram of this is shown in Figure 5. When the SWIR incident photons interact with the InGaAs two-dimensional array, the charges are generated and then converted into voltage under the control clock on the ROIC. Finally, the signal is used to create an image on the off-chip electronics. However, the smaller bandgap of the InGaAs materials produces a higher dark current, requiring the InGaAs cameras to be cooled deeply to reduce the noise level.

One factor to consider while utilizing InGaAs-based focal plane arrays is that there is a reduction in their long-wavelength cutoff after being cooled. A known rule to follow when using an InGaAs image sensor is that the long-wavelength cutoff shifts by around 8 nm for every 10 °C the sensor is cooled by [54]. This also allows the InGaAs sensor to act as a “tunable” lowpass filter. Another consideration for InGaAs sensors is that because of their complex fabrication process, practically every InGaAs focal plane array has a small number of defective pixels, such as non-uniform, dark, and bright pixels. Non-uniform pixels are pixels that show either greater or lesser photosensitive response compared to surrounding pixels. Dark pixels, on the other hand, are pixels that are unresponsive to light and appear black in the image. Finally, bright pixels are the opposite of dark pixels, wherein a pixel saturates independently of incident light and appears white in the image. Although an image sensor will have defective pixels no matter its quality, it is still typically recommended to use a defect-correction algorithm with the camera’s software or hardware to reduce the number of defective pixels as much as possible. In addition, the InGaAs cameras have limited resolution and high cost compared to other types of cameras due to their specialized manufacturing processes and small market demand. However, advances in semiconductor fabrication technology and the growing demand for InGaAs cameras will lead to increased image resolution and quality with an affordable price. InGaAs sensors are widely used in SWIR imaging systems for in vivo imaging of the blood vascular system [55], lymphatic system [56], and tumors [57].

#### 2.2.2. HgCdTe (MCT)

**Figure 6:**
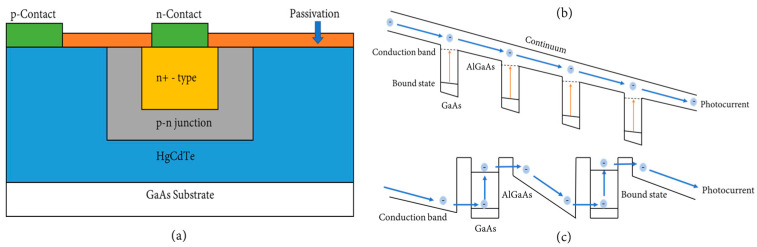
(**a**) An MCT sensor consists of a GaAs substrate, HgCdTe material, and a p−n junction. (**b**) QWIP Image Sensor and (**c**) QD Image Sensor. QWIP and QD sensors consist of a conduction band, a GaAs substrate, and an AlGaAs substrate. Electrons will move throughout the sensors as a photocurrent (blue arrows) in the continuum until they exit into a new QW/QD, or they circulate into the same QW/QD. A dark current is shown (yellow arrows) through a process called photoemission. Eventually, the photocurrent will enter a collection device for measurement or to form an image.

A commonly used sensor in the IR window is MCT. The MCT sensor is an older sensor that still plays a relevant role in IR detection even today [58,59]. MCTs can be operated in different forms including a photoconductor, a photodiode, and a semiconductor [59]. The MCT sensor can contain electrons present within it that move between its two bands, the valence band and the conduction band, when light is shone on the sensor, as shown in Figure 6a. The electrons contained in the conduction band generate an electrical current that is proportional to the amount of light shone on the sensor. The advantages of MCTs include the ability to operate at higher operating temperatures, high mobility of electrons, low dielectric constant, and high quantum efficiency. The disadvantages of MCTs are that they have bulk, surface, and interface instabilities as well as uniformity and yield issues [59]. They are also sensitive to temperatures and require cooling with liquid nitrogen to prevent noisy signals from appearing [60]. The reason MCT sensors are still used today even when various other sensors are being developed is because they are extremely flexible in that they can be used in any region of the IR spectrum, including the NIR and SWIR regions [61], meaning they can be used in a wide variety of applications.

#### 2.2.3. Quantum Well Infrared Photodetectors (QWIPs)

QWIPs are photoconductors that are known for several factors including a high impedance, a fast response time, a long integration time, and a low power consumption [60]. Compared to MCTs, a QWIP has more uniformity, yield, and a better cost when manufactured [62]. QWIPs can generally come in two forms, which include bound-to-continuum QWIPs and miniband transport QWIPs. In bound-to-continuum QWIPs, as shown in Figure 6b, the photoelectron can escape from the quantum well to the continuum transport states without having to tunnel through any barriers [58]. This reduces the voltage bias necessary to efficiently collect the photoelectrons, which also reduces the dark current produced by the sensor. The miniband transport QWIP operates by letting IR radiation be absorbed into the doped quantum wells. This excites the electron within the miniband, which then continues to transport itself until it is collected or recaptured into another quantum well. Due to a lower mobility, miniband QWIPs show a lower photoconductive gain compared to bound-to-continuum QWIPs. Among the materials used to make QWIPs, both GaAs and AlGaAs QWIPs are the most mature and well known because of the almost perfect natural lattice match between GaAs and AlGaAs. The advantages of using GaAs/AlGaAs QWIPs are that they can be utilized together with other GaAs-based technology, they are highly uniform, produce a high yield at a low cost, have a high thermal stability, and have an extrinsic radiation hardness. The main disadvantages when using QWIPs are their relatively low quantum efficiency (<10%) and their low operating temperature [59]. These factors cause QWIPs to typically be used in medical imaging systems [63].

#### 2.2.4. QD Detectors 

QD detectors can be considered an extension of QWIP detectors with additional features that can increase their performance. Similar to QWIPs, QD detectors use optical transitions between bound states in the conduction and valence band within quantum dots [64], as shown in Figure 6c. When photons shine on the QD detector, photo-excited carriers are released from a QD, and then collected as a photocurrent. Once these carriers are in the continuum, they can experience three different processes. First, the carriers can continuously drift in the continuum because of an electric field. Secondly, the carrier can be captured in the excited state of either the same QD it was released from or a separate QD. Thirdly, the carrier can be collected by a separate device for either measurement or to form an image. One of the advantages of QD detectors is that they have various material options that can change the properties of the detector [65]. The most common material used to make QDs are InAs/GaAs, where InAs QDs are embedded into GaAs through a process called epitaxial overgrowth. Other popular materials used to make QDs include AlGaAs, which can help reduce dark current, and InGaAs/Ga/As QDs, which have demonstrated IR responses in multiple wavelengths. Other advantages of QD detectors include an inherent sensitivity to light, a low dark current, and a theoretically high operating temperature (>150 k) [65]. The high operating temperature is only theoretical due to the epitaxial growth mechanism of QDs. The process causes random changes in dot size and material composition, which cause non-uniformity in the QDs. This non-uniformity causes an increase in dark current and reduces its operating temperature, which consequently reduces QD detectors’ performance. Hence, a poor performance when using a QD detector is usually caused by either the non-uniformity in the QDs or by the detector having a non-optimal band structure [64]. QD detectors are generally used in applications that involve SWIR photodetection [66,67].

### 2.3. FLIM-Based Image Sensors

Fluorescence intensity measurements can be affected by fluorophore concentration and quenching effects. Fluorescence-Lifetime Imaging Microscopy (FLIM) offers a more robust alternative to intensity-based methods for molecular imaging. FLIM employs high-speed sensors, such as Photomultiplier Tubes (PMTs), Single-Photon Avalanche Diodes (SPADs), and streak cameras to accurately capture the fluorescence decay process.

#### 2.3.1. PMT-Based Sensor

**Figure 7:**
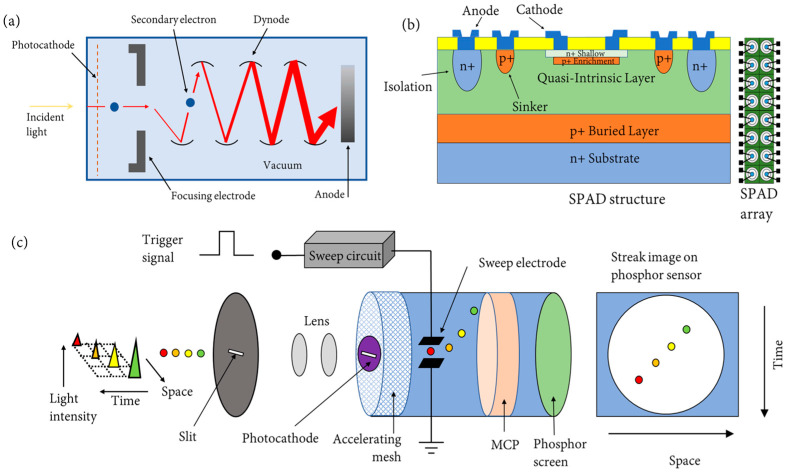
(**a**) A PMT sensor consists of a vacuum tube that contains a set of dynodes, a photocathode, a pair of focusing electrodes, and an anode. When the incident light/photon hits the photocathode, the electron will bounce off each dynode while simultaneously increasing the number of electrons (represented by the size of the red arrow) that hit the subsequent dynode. (**b**) A SPAD array consists of tens to hundreds of SPAD pixels. The SPAD pixel consists of a photodiode that can create an electron–hole pair when a photon is detected on its photosensitive side. (**c**) A streak camera contains a photocathode, an accelerating electrode, a sweep electrode, an MCP, and a phosphor screen. When the incident light with different intensity and locations (represented by the different color triangles and spots) hits the photocathode through a slit, electrons are generated based on the intensity of the light. The electrons are then accelerated by the accelerated electrode, then deflected by a pair of sweep electrodes. The electrons then pass through the MCP and hit the phosphor screen, which generates a streak image. The image can be enhanced through either a CCD or image intensifier.

A PMT is a type of FLIM sensor that is composed of a single photocathode, a dozen discrete dynodes, and an anode, as shown in Figure 7a. When a photon hits the photocathode, an electron will be released into a dynode. The collision between the electron and dynode causes several new electrons to be released, which then accelerate towards other dynodes. This repeats several times, which allows the PMT to produce an electron gain of approximately 10^6^, which yields an easily measurable current at the anode or a pulse [68]. The advantages of PMTs include a fast rise time (1–2 ns), a wide linear dynamic range, and a low dark current. The disadvantages are that they require a high-voltage-stabilized power supply, need high cooling, similar to an InGaAs sensor, require shielding from overexposure to light and magnetic fields in order to prevent burnout, and have high spatial sensitivity [68]. PMT sensors are typically used in the wide-field time-correlated single photon counting (TCSPC) method for FLIM [69,70].

#### 2.3.2. SPAD-Array Sensor

A SPAD is a FLIM sensor that consists of a photodiode whose p–n junction is reverse-biased above its breakdown voltage, which can create an electron–hole pair when a photon is detected on the photosensitive/active side of the SPAD [68,71]. This causes an “avalanche” of secondary carriers, which can be used to measure the time of photon arrival. A SPAD pixel architecture can be divided into three types based on their functionality [71]. The first pixel type only includes the basic structure of a SPAD, consisting of the circuitry necessary for a full detection cycle, which includes avalanche generation, quench, and recharge. The output of the first type of pixel is a train of electrical pulses corresponding to individual photon detections. The second pixel type includes a built-in counter, which is composed of a counting circuit and at least one bit of memory. The output of the second pixel type is a photon count. The third pixel type is time-dependent and consists of circuity that can determine the arrival time of photons, while its output can either be as simple as a flag for detection during a specified time period or as complex as a variable number of timestamps, each showing a unique photon arrival time.

SPAD sensors can also be placed into an array for imaging and used in conjunction with CMOS technology, as shown in Figure 7b. The common factors that all SPAD arrays contain include active quenching, recharging, masking, and gating. SPAD arrays can either be designed for passive or active quenching. In passive quenching, a large resistor that is placed in series to the photodiode quenches the avalanche, while in active quenching, an electronic circuit is triggered by the avalanche current to stop it. Active quenching and recharging are used to optimize the detection cycle of the SPAD array by reducing the amount of dead time, thus improving the maximum count rate [71]. Masking is used to disable specific pixels within the array. This is used to avoid the overloading that can be caused by a particular noisy pixel, which would otherwise lower the overall performance of the sensor. Gating consists of enabling the SPAD array for limited windows of time, typically within picoseconds. Gating can be used to exclude parts of the photon response that are not of interest and instead focus on the photons that are relevant.

Important factors to consider when using a SPAD sensor include its photon detection probability, dark count rate, and after-pulsing. The photon detection probability symbolizes the probability of an avalanche occurring in response to photon absorption. The dark count rate represents the observed rate in which avalanches occur in a SPAD in the absence of light, while the after-pulsing represents the number of false events that correlate in time with previous detections. The SPAD-array sensors are usually used in high-speed and dynamic FLIM studies [72,73].

#### 2.3.3. Streak Cameras

Streak cameras operate differently compared to other FLIM sensors, and they are considered the fastest sensors available. Streak cameras can typically be divided into either simple or complex streak cameras depending on how they operate [74]. The simplest streak camera uses some form or method to deflect an incident light beam, such as a rotating mirror. While the design is simple, it is also limited in terms of temporal resolution because the speed of the device is limited. A complex streak camera is typically made within a vacuum tube, as shown in Figure 7c, and is designed to convert fast time intervals into differences in geometry. It operates when light hits the photocathode within the vacuum tube, which generates a number of electrons proportional to the intensity of the light. Electrons emitted by the cathode are accelerated by a high voltage that forms a pulsed electron beam. The electron beam is deflected by a pair of electrodes, then hits a phosphor screen to generate a visible streak image, which can be recorded by a CCD camera.

The advantages of using streak cameras, in addition to their fast sensing, include the ease of handling data and that the data can typically be used for detailed analysis [75]. The disadvantage of streak cameras is that they are only capable of measuring one point at a time because one spatial dimension is used only for the time axis, while the other dimension is used only for spectral detection [68]. Streak cameras have been applied for recording multispectral FLIM data [76,77,78] and compressed ultrafast photography (CUP) for FLIM measurement [79].

## 3. Imaging Performance Comparison of Image Sensors

NIR and SWIR fluorescence imaging systems mainly consist of an excitation light source to excite fluorescent probes or dyes, a collection filter to separate the weak fluorescence signals from strong backscattered excitation light, and an image sensor to sense the fluorescence signals that are transferred to a computer for image processing and display. While the excitation light source and filtration scheme also impact the measurement sensitivity of the imaging system, we chose to assess the impact of image sensors only, with all other design features held constant. Table 1 summarizes the performance factors of each sensor.

**Figure 8:**
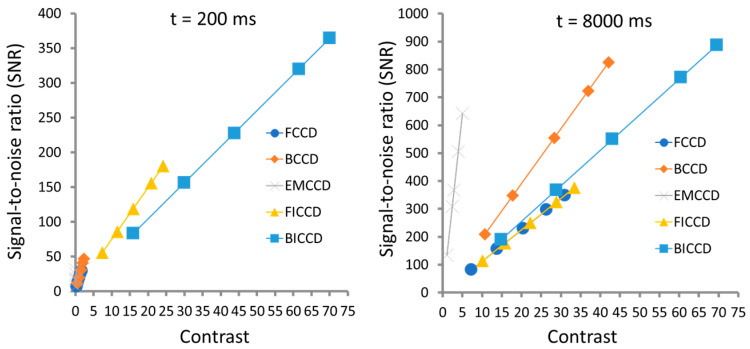
Plots of signal-to-noise vs contrast of different CCD cameras under the integration times of 200 ms and 8000 ms, respectively. Adapted from Zhu et al. [19].

Figure 8 illustrates the plots of the signal-to-noise ratio (SNR) as a function of contrast under integration times of 200 ms and 8000 ms [19]. It shows that at the short, 200 ms, integration times, ICCD camera-based NIR fluorescence imaging systems give a much higher SNR (~6 times) and contrast (~13 times) compared to their corresponding CCD camera systems, while EMCCD camera systems give the lowest SNR and contrast. This is not only because the phosphor screen in the intensifier results in the blue-shifted wavelengths, but also due to the light amplification of the MCP and the reduction in random noise through integration that occurs with the CCD camera. With longer integration times (8000 ms), the performance of unintensified CCD and EMCCD cameras improves and becomes comparable with that of intensified CCDs.

**Figure 9:**
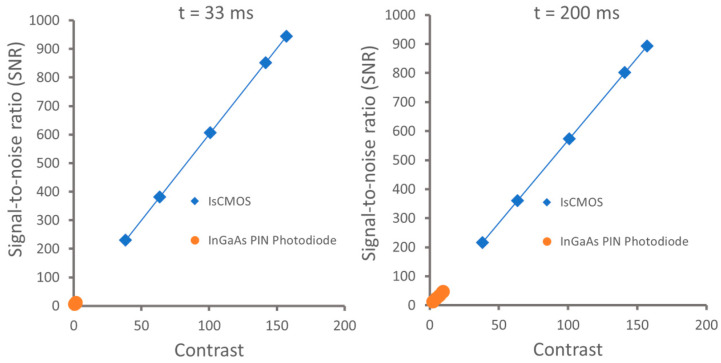
Plots of signal-to-noise vs contrast of IsCMOS camera and InGaAs PIN Photodiode under integration times of 33 ms and 200 ms, respectively. Adapted from Zhu et al. [23].

Figure 9 shows that IsCMOS-based NIR fluorescence imaging systems give much higher SNRs and contrast than those of InGaAs PIN Photodiode-based SWIR systems despite the fact that the radiance in the SWIR fluorescence channel is 2–3 orders of magnitude greater [23]. The SNR and contrast are improved at longer integrating times of 200 ms as compared to that at 33 ms for InGaAs systems. However, there are no significant improvements in both SNR and contrast at the integrating time of 200 ms for the IsCMOS system, and this is because shorter integrating times are compensated by the increased gain of the intensifier. Although the SWIR fluorescence imaging of ICG may have superior resolution in small animal imaging compared to NIR fluorescence imaging, it is the lack of measurement sensitivity, SNR, contrast, as well as water absorption that limits deep penetration in large animals.

## 4. Conclusions and Outlook

In this paper, we have presented the different types of image sensors used in NIR and SWIR fluorescence imaging systems, including Si-based image sensors, such as CCD cameras, EMCCD cameras, and CMOS cameras, and GaAs-based intensified image sensors, such as ICCD cameras and ICMOS cameras, and InGaAs-, MCT-, QWIP- and QD-based area detectors. Their architecture, noise factors, and imaging performance have been discussed and compared. Since each type of image sensor has its own advantages and disadvantages, one can choose the right type of image sensor based on their specific applications. For example, an ICCD camera is the best choice for real-time, video-rate imaging, while EMCCDs and non-intensified cameras may be more suitable when fluorescent dye is available at higher concentrations than is typical for molecular imaging applications or when long image acquisition times can be tolerated.

Due to the relative maturity of NIR sensors, SWIR sensors face more challenges that require improvement for better performance when used in applications. SWIR sensors have a much higher dark current and require the need for very deep cooling due to a lower bandgap [60]. SWIR detectors also have a relatively time-consuming and difficult manufacturing process compared to NIR sensors, making SWIR sensors more expensive. For example, combining the InGaAs array with a readout circuit is considered complex and time-consuming because of the many steps required for the process. Another challenge is that each pixel in a SWIR sensor shows a slightly different behavior, making the raw image appear noisy [80]. The last challenge that SWIR sensors face is their trouble implementing a bandpass filter. Similar to NIR sensors, when a bandpass filter is used in a SWIR sensor, the filter experiences a wavelength shift as a function of the angle of incidence [80]. The center wavelength of the filter will shift towards a shorter wavelength as the viewing angle widens.

Some strategies have been proposed to resolve these issues when using SWIR sensors. For instance, some cameras that utilize SWIR sensors have a built-in sensor-cooling and temperature control system using a Peltier element, which allows the sensor temperature to remain relatively low [60]. Another solution involves including a preprocessing algorithm to correct factors such as noise generated by each pixel’s separate behavior as well as defective pixels such as dark and bright pixels [60]. To lower manufacturing costs and implement high-volume production of SWIR sensors, researchers are either developing new technology or integrating the exciting mature techniques that allow for the incorporation of electronic circuits on the same silicon chip. To improve the performance of SWIR sensors, recent studies have been exploring new materials and new structures [81]. Overall, numerous studies that involve NIR and SWIR sensors have the potential to improve both technologies and resolve some of the challenges that plague both sensors currently, such as improving the resolution of the detectors or implementing multi-band filters into the detectors.

Currently, ICG is the only FDA-approved contrast agent that has been widely used in the clinic for NIR fluorescence imaging [1]. ICG also exhibits optical properties at SWIR wavelengths, even beyond 1500 nm, and preclinical studies show real-time fluorescence imaging of blood and lymph vessels as well as hepatobiliary clearance using ICG at clinically relevant doses [18]. To accelerate the clinical translation of NIR and SWIR fluorescence imaging, the new developing fluorophores should have low toxicity, be bright, and display limited photobleaching. In addition, the imaging devices should be adopted with highly sensitive detectors, able to detect micro doses of fluorophores.

## Figures and Tables

**Table 1:** Performance factor comparison between sensors.

Image Sensor	Noise	SNR	Imaging Speed	Cost	Spectral Range	Applications
**CCD**	Low	Medium	Slow	Medium	NIR	NIR fluorescence molecular tomography; NIR fluorescence-guided surgery imaging
**EMCCD**	High	Low	Average	High	NIR	NIR fluorescence imaging of the lymphatic vasculatures in small animal models
**CMOS**	Medium	Medium	Average	Low	NIR	NIR fluorescence imaging of lymphatic system
**ICCD/** **ICMOS**	Low/ Medium	High	Fast	High	NIR	Fluorescence tomographic imaging; rapid imaging of lymphatic function
**InGaAs**	High	Medium	Average	High	SWIR	SWIR imaging of the blood vascular system, lymphatic system, and tumors
**MCT**	High	Low	Fast	High	NIR SWIR	IR spectrum including the NIR and SWIR regions
**QWIP**	High	Low	Average	Low	NIR SWIR	Medical imaging
**QD**	Low	High	Average	Low	NIR SWIR	Medical imaging; microscopy
**PMT**	Low	High	Fast	High	NIR	Wide-field TCSPC in FLIM
**SPAD** **Array**	Low	High	Fast	High	NIR SWIR	High-speed and dynamic FLIM studies
**Streak Camera**	Low	High	Fast	High	NIR SWIR	Multispectral FLIM; compressed ultrafast photography

## Data Availability

Not applicable.
